# Analysis of sternal healing after median sternotomy in low risk patients at midterm follow-up: retrospective cohort study from two centres

**DOI:** 10.1186/s13019-019-1000-1

**Published:** 2019-11-11

**Authors:** Bin Wang, Dapu He, Min Wang, Yongxiang Qian, Youran Lu, Xinping Shi, Yang Liu, Xianghong Zhan, Dongmei Di, Kai Zhu, Xiaoying Zhang

**Affiliations:** 1 Department of Cardiothoracic Surgery, the Third Affiliated Hospital of Soochow University, Juqian Street, Changzhou, 185 China; 2 Department of Cardiothoracic Surgery, the First Affiliated Hospital of Nanhua University, Hengyang, China; 3 Department of Radiology, the Third Affiliated Hospital of Soochow University, Juqian Street, Changzhou, 185 China; 4 Department of Cardiac Surgery, Zhongshan Hospital of Fudan University, Fenglin Street, Shanghai, 180 China

**Keywords:** Sternotomy, Wound healing, Bone plates, Bone wires

## Abstract

**Background:**

For low risk patients undergoing median sternotomies, no midterm follow-up studies involving sternal healing have been conducted. In this study we evaluated sternal healing in low risk patients by chest CT scan and the risk factors associated with poor healing were analyzed.

**Methods:**

Patients who underwent sternal median incision heart surgery from September 2014 to March 2015 were recruited. The clinical information of these patients during hospitalization was collected, and the CT scan data were submitted to the two chief physicians of the Radiology Department for radiographical sternal healing score determination. Based on the method of wound closure, the patients were divided into sternum plate (Plates) and wire groups (Wires).

**Results:**

Forty-four patients were recruited. The mean CT examination time was 17.27 ± 2.30 months postoperatively. Twenty-nine (65.9%) patients met the criteria for radiographic sternal healing. Three segments, including the aortopulmonary window, the main pulmonary artery, and the aortic root, had healed less in comparison to the manubrium segment. Compared to patients in whom 6–7 metal wires were used for sternal closure, healing of the lower sternum was worse in patients in whom five wires were used, but the difference in healing was not statistically significant. Univariate analysis of sternal healing showed that patient age was a risk factor for sternal non-healing. When the patient age was > 45 years, the predicted risk of radiographic sternal non-union was 1.833 (95% CI: 1.343–2.503).

**Conclusions:**

At the mid-term follow-up, 65.9% of patients undergoing median sternotomies demonstrated radiographic sternal healing. Age, but not closure device, was a risk factor for sternal non-healing in low risk patients. Use of more wires had a positive impact on sternal healing.

**Trial registration:**

researchregistry4918, registered 28 May 2019, retrospectively registered.

## Background

A sternal median incision is a commonly used incision for heart and major vascular surgery. This method has been used for a long time; however, there is a 0.8–1.5% probability of sternal dehiscence or mediastinal infection after surgery [1]. The probability of sternal dehiscence or mediastinal infection could be as high as 2.4% after bilateral internal mammary artery grafting, thus leading to multiple complications and even death [2]. Shin reported that 34.5% of patients had poor sternal healing 3–6 months after surgery. Risk factors for poor sternal healing included advanced age, diabetes, and post-operative renal insufficiency. In a long-term follow-up, 98.2% of patients achieved healing based on imaging assessment [3]. It is apparent that sternal healing is a long-term process. With respect to low risk patients undergoing median sternotomies, no midterm follow-up studies involving sternal healing have been conducted. In this study we excluded the interference of most risk factors and evaluated sternal healing in low-risk patients based on chest computerized tomography (CT) scan and analyzed the risk factors associated with poor healing.

## Methods

### Patients

In April 2016, patients who underwent sternal median incision heart surgeries in the Department of Cardiothoracic Surgery at the Third Affiliated Hospital of Soochow University and the First Affiliated Hospital of Nanhua University from September 2014 to March 2015 were recruited. The inclusion criteria were as follows: 1. between 18 and 70 years of age; and 2. cardiac surgery with a total sternal median incision. The exclusion criteria were as follows: 1. history of a sternotomy; 2. peri-operative history of immunosuppression drug or hormone use; 3. history of diagnosis of autoimmune disease; 4. history of bone metabolism- or bone development-related diseases; 5. history of severe renal insufficiency (preoperative creatinine > 200 μmol/L); 6. bilateral internal mammary artery-free surgery; 7. second thoracotomy after surgery; and 8. post-operative treatment with intra-aortic balloon pump or continuous renal replacement therapy. The patients or their families were contacted by telephone to encourage the patients return to the hospital for an examination and chest CT scan to assess sternal healing. Forty-four patients (23 from the Third Affiliated Hospital of Soochow University and 21 from the First Affiliated Hospital of Nanhua University) were recruited and all patient data were used for analysis.

### Analysis of sternal healing

**Fig. 1:**
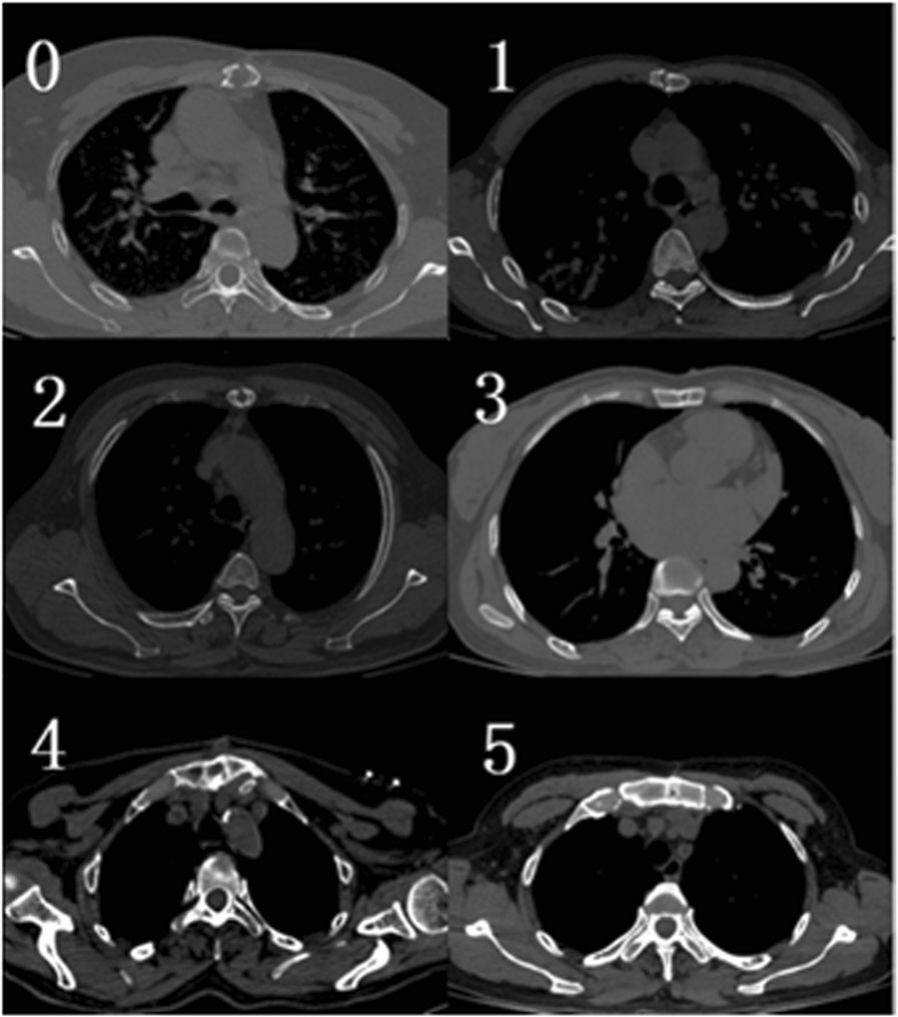
Axial computed tomography scan images representative of each score on 6-point scale. Each score was defined as follows: (0) no sign of healing (clear lack of healing, nonunion, sternal separation); (1) minimal healing (sternal separation, lack of bridging bone); (2) mild healing (sternal separation with hazy, immature bone formation); (3) moderate healing (partial bridging bone indicative of sternal stability); (4) partial synthesis (significant bridging bone); (5) complete synthesis (complete bridging bone)

**Fig. 2:**
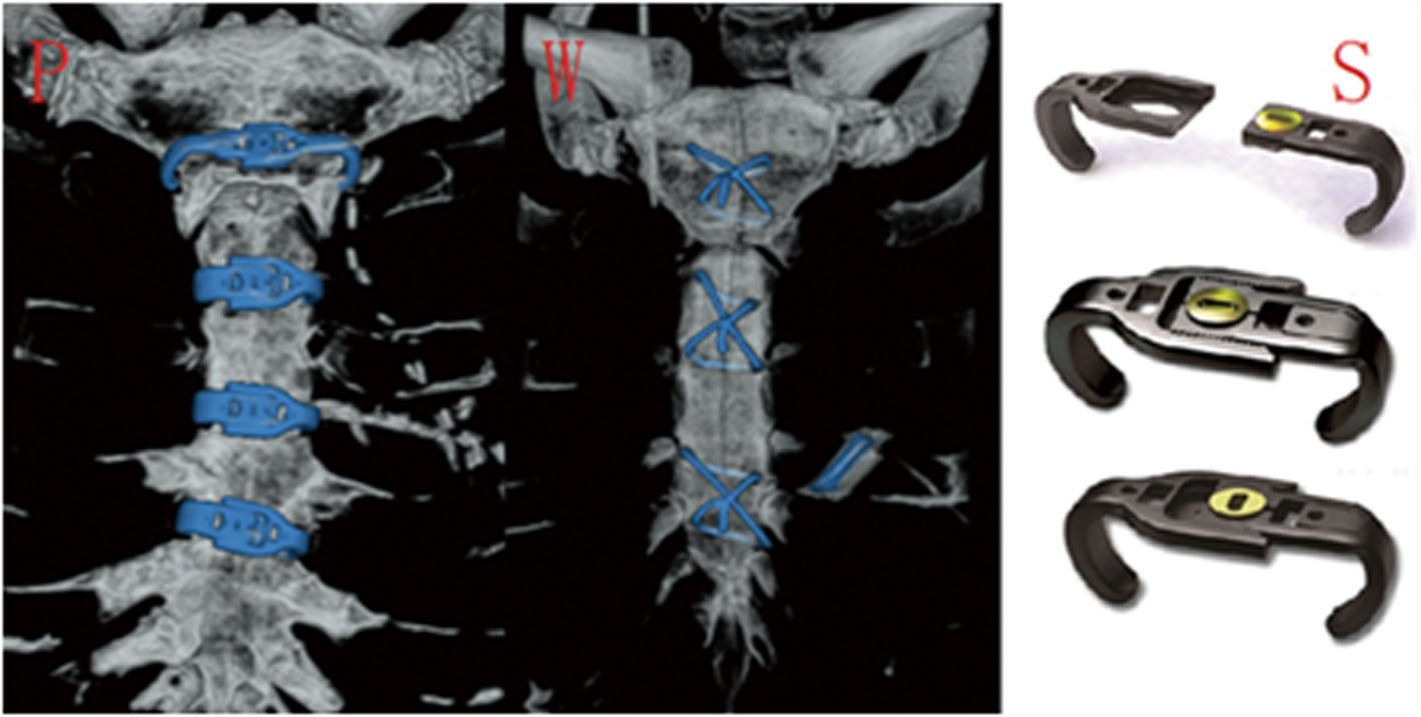
3D reconstruction of sternum after median sternotomy. (P): closed using titinium plates; (W): closed using wires

Clinical information was collected from the 44 patients during hospitalization, and the CT scan data were submitted to the two chief physicians of the Radiology Department of the Third Affiliated Hospital of Soochow University, who determined the radiographic sternal healing scores. The scoring method was based on Raman [4]. Axial slices were analyzed at five locations along the sternum (manubrium, top of the aortic arch, aortopulmonary window, main pulmonary arteries, and aortic root) using a 6-point quantitative scale (0: no sign of healing, 1: minimal healing, 2: mild healing, 3: moderate healing, 4: partial synthesis, 5: complete synthesis; Fig. 1). Sternal union was defined as a mean score > 3. The sternum was closed by one of the two following methods: titanium plates (Sternal Fixation System; Waston Medical Appliance Co., Ltd.), in which four plates are used in the first, second, third, and fourth or fifth intercostal spaces; and closure of the sternum with 5–7 metal wires (Surgical Stainless Steel Suture; Ethicon, LLC) as a single interrupted suture or a figure-of-eight suture (Fig. 2). On the method of closure, the patients were divided into sternum plate (Plates) and wire groups (Wires). All patients were given non-absorbable bone wax for sternal hemostasis. This work has been reported in line with the STROCSS criteria [5].

### Statistical analysis

The sternal healing score was tested by a paired *t*-test to determine the repeatability and reliability of the scoring method. Continuous variable data were analyzed by an independent *t*-test. Categorical variable data were analyzed by a chi-square test. A ROC curve was used to assess the value of risk factors for predicting sternal healing.

## Results

### Paired *t*-test for the radiographic sternal healing score method

The mean CT examination time was 17.27 ± 2.30 months (range, 13–21 months) post-operatively. The two imaging chief physicians determined the healing score based on the sternal CT scan. The results were analyzed by a paired *t*-test. The results showed that the scoring method was reliable and repeatable (Table 1).

**Table 1:** Paired *t*-test for the radiographic sternal healing score method

	T score	*P* value
Manubrium	0	1.000
Top of aortic arch	1.159	0.253
Aortopulmonary window	0.553	0.583
Main pulmonary artery	0.404	0.688
Aortic root	−0.813	0.420

### Peri-operative characteristics and radiographic sternal healing score

**Fig. 3:**
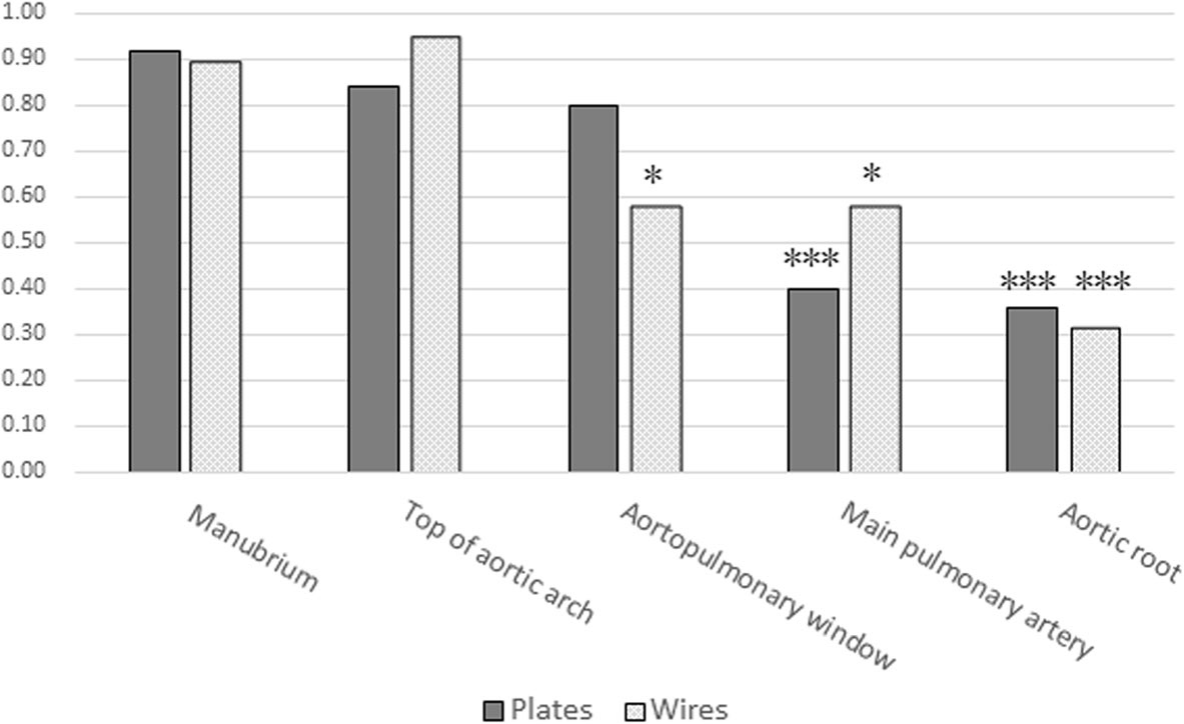
Radiographic healing of the five segments of sternum. ^*^P < 0.05, ^***^P < 0.001

Forty-four patients were recruited, including 23 males and 21 females, with an average age of 51.2 ± 12.3 years. The mean hospital stay was 26.8 ± 10.3 days, and the mean postoperative hospital stay was 18.1 ± 6.9 days. The mean body weight was 61.6 ± 11.3 kg. Nine and 36 patients underwent unilateral internal mammary artery-free and extracorporeal circulation, respectively. Isolated coronary artery bypass graft surgery was performed in nine patients, isolated valve procedures in 29 patients, and combination coronary artery bypass graft/valve procedures in one patient. On the first day postoperatively, the mean drainage volume was 437.6 ± 247.8 mL. On the second day postoperatively, the mean drainage volume was 210.2 ± 191.0 mL. The mean total drainage volume was 772.7 ± 480.8 mL. The mean time for removal of the chest tube was 3.8 ± 1.1 days. The mean postoperative ventilator maintenance time was 19.2 ± 8.9 h. Twenty-three patients required postoperative infusions of an erythrocyte suspension or blood plasma. An erythrocyte suspension (1.3 ± 2.3 U) and blood plasma (286.4 ± 516.4 mL were infused. The mean sternal healing score was 3.3 ± 0.9. Twenty-nine (65.9%) patients met the criteria for radiographic sternal healing. The healing rate was 40/44 at the manubrium segment, 39/44 at the top of aortic arch segment, 31/44 at the artero-pulmonary window segment, 21/44 at the main pulmonary artery segment, and 15/44 at the aortic root segment (Table 2). Three segments (artero-pulmonary window, main pulmonary artery, and aortic root) were less healed in comparison to the manubrium (Fig. 3). No patients complained of sternal-related symptoms and had good life and social skills.

**Table 2:** Clinical characteristics and sternal healing in different sternal closure groups

	Plates(N = 25)	Wires(N = 19)	P value
Age (year)	48.2 ± 13.2	55.2 ± 10.0	0.06
Male	10/25	13/19	0.06
Weight (Kg)	60.6 ± 13.2	63.0 ± 8.3	0.51
BMI (Kg/m^2^)	22.73 ± 4.35	22.97 ± 2.36	0.83
Unilateral internal mammary artery free	4/25	5/19	0.40
Extracorporeal circulation	20/25	16/19	0.72
Drainage on the first day (mL)	450.0 ± 280.1	421.3 ± 203.9	0.68
Total Drainage (mL)	721.0 ± 397.6	840.8 ± 576.9	0.32
Chest tube removal time (day)	3.7 ± 1.0	4.0 ± 1.3	0.41
Postoperative ventilation time (hour)	18.5 ± 8.6	20.1 ± 9.4	0.85
Sternal healed	18/25	11/19	0.33
Manubrium segment healed	23/25	17/19	1.00
Top of aortic arch segment healed	21/25	18/19	0.37
Aortopulmonary window segment healed	20/25	11/19	0.18
Main pulmonary artery segment healed	10/25	11/19	0.36
Aortic root segment healed	9/25	6/19	1.00

### Comparison of plates and wires groups

An independent *t*-test did not show a difference between the Plates group (*n* = 25) and Wires group (*n* = 19) with respect to age, length of post-operative hospital stay, weight, body mass index (BMI), pre-operative blood test results, post-operative drainage, chest tube removal time, post-operative ventilator assist time, blood infusion, and sternal healing (Table 2). A subgroup analysis was performed on data of the patients in the Wires group. Compared to the healing results in patients in whom 6–7 metal wires were used for sternal closure, sternal healing of the lower sternum in patients in whom five wires was used was worse, but the difference was not statistically significant (*P* = 0.096) (Table 3). In addition, we found that the patients using five wires weighed less and had significantly decreased drainage.

**Table 3:** Subgroup analysis of sternal healing in the Wires group

	6–7 wires(*N* = 9)	5 wires(*N* = 10)	P value
Weight (Kg)	66.67 ± 9.49	59.60 ± 5.7	0.063
BMI (Kg/m^2^)	23.50 ± 2.78	22.50 ± 1.95	0.37
Drainage on the first day (mL)	322.50 ± 188.73	531.11 ± 166.47	0.021
Total Drainage (mL)	470.50 ± 317.48	1252.22 ± 523.02	0.001
Sternal healed	7/9	4/10	0.096
Manubrium segment healed	8/9	9/10	1.00
Top of aortic arch segment healed	9/9	9/10	1.00
Aortopulmonary window segment healed	6/9	5/10	0.79
Main pulmonary arterysegment healed	6/9	5/10	0.79
Aortic root segment healed	4/9	2/10	0.52

### Risk factors for sternal healing

**Fig. 4:**
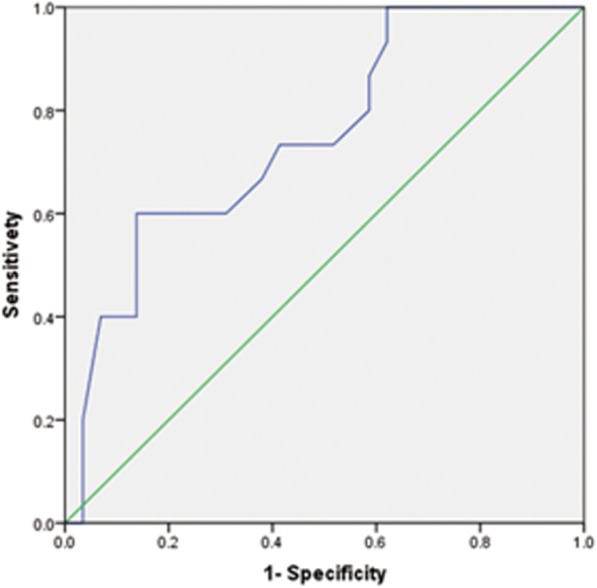
ROC analysis of age prediction for radiographic sternal nonunion

Univariate analysis of sternal healing showed that patient age was a risk factor for sternal non-healing, unlike diabetes, smoking history, unilateral internal mammary artery grafting, extracorporeal circulation, and post-operative drainage (Table 4). The area under ​​the ROC curve (AUC) for age as a predictor for radiographic sternal non-union was 0.748 and the cut-off value was 45 years (Fig. 4). At an age > 45 years, the predicted risk of radiographic sternal non-union was 1.833 (95% CI: 1.343–2.503).

**Table 4:** Univariate analysis of radiographic sternal healing

	Healed(*N* = 29)	Unhealed(*N* = 15)	P value
Sternal healing score	3.73 ± 0.51	2.32 ± 0.63	0.000
Age	47.62 ± 12.45	58.13 ± 8.68	0.006
Male	17/29	6/15	0.241
Weight	61.62 ± 10.92	61.67 ± 12.40	0.990
BMI (Kg/m^2^)	22.51 ± 3.26	23.47 ± 4.22	0.40
Diabetes	3/29	0/15	0.197
Smoking history	2/29	3/15	0.194
Unilateral internal mammary artery free	6/29	3/15	0.957
Extracorporeal circulation	24/29	12/15	0.822
Off-midline sternotomy	3/29	2/15	0.77
Sternal closure with titinium plates	18/29	7/15	0.328
Drainage on the first day	456.90 ± 266.15	400.33 ± 211.29	0.479
Total drainage	755.34 ± 482.55	767.67 ± 494.13	0.961
Postoperative ventilation time (hour)	19.79 ± 9.24	18.07 ± 8.38	0.548
Follow-up time (month)	17.55 ± 2.03	16.73 ± 2.74	0.267

## Discussion

Although the incidence of sternal-related complications after sternotomy is low, the mortality rate is as high as 47% [6], and good sternal healing is closely related to a good quality of life [7]. Schimmer [8] reported that BMI > 30 kg/m^2^, heart function greater than NYHA III, renal insufficiency, peripheral arterial disease, an immunosuppressive state, surgical assistant closure of the chest, postoperative bleeding, infusion of plasma-reduced blood > 5 U, secondary surgery hemostasis, and surgery posterior malleous were risk factors for post-operative sternal complications. In addition, age > 42 years, a history of sternal surgery, > 2 arterial grafts (> 2 arterial conduits), internal mammary artery-free, BMI, chronic heart failure, diabetes, respiratory failure, and unexpected secondary surgery could lead to sterile sternal dehiscence [9]. A meta-analysis also indicated that a number of risk factors for sternal complications (OR values: 1.98 for female gender, 1.28 for smoking, 3.31 for diabetes, 2.59 for obesity, 3.11 for bilateral internal mammary artery grafting, 8.92 for secondary surgery, and 2.84 for transfusion) [6]. Furthermore, off-midline sternotomy was the cause of sternal dehiscence [10].

Regarding the challenges of sternal complications, many researchers are working to reduce the occurrence. A cadaveric experiment performed by McGregor [11] confirmed that displacement of the xiphoid end under pressure is greater than the manubrium end. Dasika [12] also showed that the lower sternum is the most unstable site of the sternum in artificial sternal models. Additional reinforcement of the site could enhance sternal stability, and there was no difference between a figure-of-eight wire closure and a single wire closure. In addition, double-strand steel wires close the sternum with higher stability than single steel wire [13]. The cadaveric experiment suggested that reinforcement of sternal wires with stainless steel coils improves sternal stability [14]. Prophylactic sternal weave closure reduces the risk of postoperative sternal dehiscence in patients with diabetes-related obesity and concurrent coronary artery bypass surgery [15]; however, prophylactic sternal weave closure did not present an advantage for patients with one risk factor [8]. With the development of science and technology, a variety of new sternum closure devices can significantly improve sternal healing. The early sternal healing rate is significantly improved, while the cost is not dramatically increased, including sternum plates and thermo-reactive nitrilium clips, which are quite important in high-risk patients [16, 17]. For low risk patients, the sternal plate did not present values in hospital stay, cost, and incidence of sternal complications [18]. In addition, a thoracic support vest was beneficial for healing the sternum, and also reduced mechanical sternal complications and shortened the hospital stay [19].

After the sternal incision, there is more bleeding from the inner and outer periosteum and bone marrow. Bone wax is conventionally used to control bleeding, but absorption of non-degradable bone wax is difficult for patients. Water-soluble polymer wax is more beneficial to sternal healing than bone wax [7].

As part of the sternum, the role of the xiphoid cannot be overlooked. In comparison to a traditional median sternotomy, a xiphoid-sparing median sternotomy is effective in reducing the risk of wound and mediastinal infections [20]. Intramedullary incarceration of a cancellous portion of the autologous xiphoid can also promote healing of the sternum and reduce the probability of pseudo-joint formation [21].

Platelet-rich plasma can promote bone healing [22]. Shibata [23] reported that controlled release of platelet-rich plasma from locally-applied gelatin hydrogel improves early sternal healing in patients undergoing sternotomies. Intra-sternal injection of autologous platelet-rich plasma also reduces the incidence of sternal complications and reduces the probability of rehospitalization [24].

Loosening of the sternum may lead to sternal dehiscence, and secondary incision and mediastinal infections. Mediastinal infections or sternal defects usually require surgery for debridement, drainage, and reconstruction [25]. For patients with a loosened sternum who do not need surgery or cannot undergo sternal reconstruction, low level laser therapy could be used for the treatment of upper sternal loosening, and trunk stabilization exercises are more suitable for the treatment of lower sternal loosening [26].

In this study we found that the mid-term sternal union proportion after sternotomy was 65.9%, which was unexpected, especially because the sternal union proportion reached 98.2% between 24 and 48 months after surgery [3]. Inferior to the manubrium, the healing rate of the sternal segments gradually decreased, suggesting that poor healing mainly occurred in the lower sternum, which was consistent with the findings from in vitro experiments [11, 12]. Given that our subjects were low risk patients, no difference in healing scores existed between the Plates and Wires groups, and the results were consistent with the report by Peigh [18]. In the subgroup analysis of the Wires group, we found that by using 6–7 wires for closure, the sternal healing rate was slightly better than the use of five wires for closure, suggesting that increasing the number of suture wires improved sternal healing, especially in the lower sternum. Univariate analysis suggested that patient age > 45 years was an independent risk factor for poor sternal healing, which was consistent with other studies. Other risk factors were not identified due to the small sample size in this study.

There were several limitations in this study. First, the sample size was small and no other risk factors, including diabetes, smoking, and obesity, were identified. Second, the study only analyzed low risk patients, while high risk patients could have worse sternal healing in the midterm stage. Third, 10 patients in the study only received five wires for sternal closure, which was not up to the standard and affected the results of sternal healing. We expect a study with a larger sample size to assess the progress of sternal healing after sternotomy.

## Conclusions

At the midterm follow-up evaluation, 65.9% of patients undergoing median sternotomies demonstrated radiographic sternal healing. Age, but not closure devices, was the risk factor for sternal nonhealing in low risk patients, and use of more wires was beneficial for sternal healing.

## Data Availability

The datasets used and/or analyzed during the current study are available from the corresponding author on reasonable request.

## Abbreviations

BMI: Body mass index
CT: Computerized tomography

## References

[1] Kaul P (2017). Sternal reconstruction after post-sternotomy mediastinitis. J Cardiothorac Surg.

[2] Bonacchi M, Prifti E, Bugetti M, Parise O, Sani G, Johnson DM (2018). Deep sternal infections after in situ bilateral internal thoracic artery grafting for left ventricular myocardial revascularization: predictors and influence on 20-year outcomes. J Thorac Dis.

[3] Shin YC, Kim SH, Kim DJ, Kim DJ, Kim JS, Lim C (2015). Sternal healing after coronary artery bypass grafting using bilateral internal thoracic arteries: assessment by computed tomography scan. Korean J Thorac Cardiovasc Surg.

[4] Shin YC, Kim SH, Kim DJ, Kim DJ, Kim JS, Lim C (2012). Sternal closure with rigid plate fixation versus wire closure: a randomized controlled multicenter trial. Ann Thorac Surg.

[5] Agha RA, Borrelli MR, Vella-Baldacchino M, Thavayogan R, Orgill DP (2017). The STROCSS group. The STROCSS statement: strengthening the reporting of cohort studies in surgery. Int J Surg.

[6] Balachandran S, Lee A, Denehy L, Lin KY, Royse A, Royse C (2016). Risk factors for sternal complications after cardiac operations: a systematic review. Ann Thorac Surg.

[7] Vestergaard RF, Nielsen PH, Terp KA, Søballe K, Andersen G, Hasenkam JM (2014). Effect of hemostatic material on sternal healing after cardiac surgery. Ann Thorac Surg.

[8] Schimmer C, Reents W, Berneder S, Eigel P, Sezer O, Scheld H (2008). Prevention of sternal dehiscence and infection in high-risk patients: a prospective randomized multicenter trial. Ann Thorac Surg.

[9] Fu RH, Weinstein AL, Chang MM, Argenziano M, Ascherman JA, Rohde CH (2016). Risk factors of infected sternal wounds versus sterile wound dehiscence. J Surg Res.

[10] Shafir R, Weiss J, Herman O, Cohen N, Stern D, Igra Y (1988). Faulty sternotomy and complications after median sternotomy. J Thorac Cardiovasc Surg.

[11] McGregor WE, Trumble DR, Magovern JA (1999). Mechanical analysis of midline sternotomy wound closure. J Thorac Cardiovasc Surg.

[12] Dasika UK, Trumble DR, Magovern JA (2003). Lower sternal reinforcement improves the stability of sternal closure. Ann Thorac Surg.

[13] Losanoff JE, Basson MD, Gruber SA, Huff H, Hsieh FH (2007). Single wire versus double wire loops for median sternotomy closure: experimental biomechanical study using a human cadaveric model. Ann Thorac Surg.

[14] McGregor WE, Payne M, Trumble DR, Farkas KM, Magovern JA (2003). Improvement of sternal closure stability with reinforced steel wires. Ann Thorac Surg.

[15] Aykut K, Celik B, Acıkel U (2011). Figure-of-eight versus prophylactic sternal weave closure of median sternotomy in diabetic obese patients undergoing coronary artery bypass grafting. Ann Thorac Surg.

[16] Allen KB, Thourani VH, Naka Y, Grubb KJ, Grehan J, Patel N (2017). Randomized, multicenter trial comparing sternotomy closure with rigid plate fixation to wire cerclage. J Thorac Cardiovasc Surg.

[17] Bejko J, Tarzia V, De Franceschi M, Bianco R, Castoro M, Bottio T (2012). Nitinol flexigrip sternal closure system and chest wound infections: insight from a comparative analysis of complications and costs. Ann Thorac Surg.

[18] Peigh G, Kumar J, Unai S, James DT, Hirose H (2017). Randomized trial of sternal closure for low risk patients: rigid fixation versus wire closure. Heart Surg Forum.

[19] Caimmi PP, Sabbatini M, Kapetanakis EI, Cantone S, Ferraz MV, Cannas M (2017). A randomized trial to assess the contribution of a novel thorax support vest (corset) in preventing mechanical complications of median sternotomy. Cardiol Ther.

[20] Rashed A, Verzar Z, Alotti N, Gombocz K (2018). Xiphoid-sparing midline sternotomy reduces wound infection risk after coronary bypass surgery. J Thorac Dis..

[21] Chang JP (2018). Intramedullary reinforcement of sternal fixation with autologous xiphoid tenon. J Thorac Dis..

[22] Kanthan SR, Kavitha G, Addi S, Choon DS, Kamarul T (2011). Platelet-rich plasma (PRP) enhances bone healing in non-united critical-sized defects: a preliminary study involving rabbit models. Injury.

[23] Shibata M, Takagi G, Kudo M, Kurita J, Kawamoto Y, Miyagi Y (2018). Enhanced sternal healing through platelet-rich plasma and biodegradable gelatin hydrogel. Tissue Eng Part A.

[24] Patel AN, Selzman CH, Kumpati GS, McKellar SH, Bull DA (2016). Evaluation of autologous platelet rich plasma for cardiac surgery: outcome analysis of 2000 patients. J Cardiothorac Surg.

[25] El Oakley RM, Wright JE (1996). Postoperative mediastinitis: classification and management. Ann Thorac Surg.

[26] Helmy ZM, Mehani SHM, El-Refaey BH, Al-Salam EHA, Felaya EEE. Low-level laser therapy versus trunk stabilization exercises on sternotomy healing after coronary artery bypass grafting: a randomized clinical trial. Lasers Med Sci. 2018. 10.1007/s10103-018-02701-4. 10.1007/s10103-018-02701-4 30547261

